# Expression of glyoxalase-I is reduced in cirrhotic livers: A possible mechanism in the development of cirrhosis

**DOI:** 10.1371/journal.pone.0171260

**Published:** 2017-02-23

**Authors:** Marcus Hollenbach, Antje Thonig, Sabine Pohl, Cristina Ripoll, Maurice Michel, Alexander Zipprich

**Affiliations:** Department of Internal Medicine I, Martin Luther University Halle-Wittenberg, Halle, Germany; Saint Louis University, UNITED STATES

## Abstract

**Background:**

High concentrations of methylglyoxal (MGO) cause cytotoxiticy via formation of advanced glycation endproducts (AGEs) and inflammation. MGO is detoxificated enzymatically by glyoxalase-I (Glo-I). The aim of this study was to analyze the role of Glo-I during the development of cirrhosis.

**Methods:**

In primary hepatocytes, hepatic stellate cells (pHSC) and sinusoidal endothelial cells (pLSEC) from rats with early (CCl_4_ 8wk) and advanced cirrhosis (CCl_4_ 12wk) expression and activity of Glo-I were determined and compared to control. LPS stimulation (24h; 100ng/ml) of HSC was conducted in absence or presence of the partial Glo-I inhibitor ethyl pyruvate (EP) and the specific Glo-I inhibitor BrBzGSHCp_2_. MGO, inflammatory and fibrotic markers were measured by ELISA and Western blot. Additional rats were treated with CCl_4_ ± EP 40mg/kg b.w. i.p. from wk 8–12 and analyzed with sirius red staining and Western blot.

**Results:**

Expression of Glo-I was significantly reduced in cirrhosis in whole liver and primary liver cells accompanied by elevated levels of MGO. Activity of Glo-I was reduced in cirrhotic pHSC and pLSEC. LPS induced increases of TNF-α, Nrf2, collagen-I, α-SMA, NF-kB and pERK of HSC were blunted by EP and BrBzGSHCp_2_. Treatment with EP during development of cirrhosis significantly decreased the amount of fibrosis (12wk CCl_4_: 33.3±7.3%; EP wk 8–12: 20.7±6.2%; p<0.001) as well as levels of α-SMA, TGF-β and NF-κB *in vivo*.

**Conclusions:**

Our results show the importance of Glo-I as major detoxifying enzyme for MGO in cirrhosis. The reduced expression of Glo-I in cirrhosis demonstrates a possible explanation for increased inflammatory injury and suggests a “vicious circle” in liver disease. Blunting of the Glo-I activity decrease the amount of fibrosis in established cirrhosis and constitutes a novel target for antifibrotic therapy.

## Introduction

Chronic liver inflammation secondary to different noxious agents can lead to the development of cirrhosis. This inflammation activates hepatic stellate cells (HSC) directly by means of endotoxin (LPS) or indirectly through proinflammatory cytokines. Activated HSC acquire a myofibroblastic phenotype which enhances collagen deposition [1] and therefore fibrosis. Myofibroblasts lead to activation of factors for cell growth, cell proliferation and cell differentiation, mainly mitogen-activated protein kinases (MAPK), and activation of transcription factors such as nuclear factor-kB (NF-kB) [2,3].

Advanced-glycation-endproducts (AGEs) are observed in several pathophysiological situations associated with inflammation and are mainly produced due to the cytotoxicity of methylglyoxal (MGO). At high concentrations, MGO reacts with proteins, nucleic acids and phospholipids leading to the generation of AGEs [4]. AGEs bind to their receptor (RAGE) leading to the activation of different signaling pathways including the MAPkinase ERK 1/2, as well as the downstream activation of nuclear factor kB (NF-ĸB) [5,6], all of which have been involved in the activation of stellate cells.

MGO is ubiquitously distributed in cells, as it is a by-product of glycolysis and the ketone bodies pathway. In order to avoid its above mentioned cytotoxicity, it is detoxified by means of the glyoxalase system. The enzymes glyoxalase I (Glo-I) and glyoxalase II (Glo-II) catalyze the conversion of MGO to the hemithioacetal S-D-lactoylglutathione using L-glutathione (GSH) as a cofactor (S1 Fig) [7].

Despite the fact that MGO and its detoxification through Glo-I have been involved in several pathways which hypothetically could lead to stellate cell activation and liver fibrosis, its implication in these phenomena and cirrhosis is unknown. Furthermore the effects of partial inhibition of Glo-I activity in hepatic stellate cells has not been explored. Therefore, the aim of the study was to investigate the expression and activity of Glo-I in early and advanced cirrhosis. A secondary aim was to evaluate the effect of Glo-I inhibition on the hepatic stellate cell secretion of proinflammatory cytokines and markers of fibrosis. Finally, we analyzed the effect of partial inhibition of Glo-I on progression of cirrhosis.

## Material and methods

### Induction of cirrhosis

Male Wistar rats (Center of Medical Basic Research (ZMG), Medical Faculty, University of Halle) were held in standard cages including 4 animals with free access to water and food in a climate room with 12-hour circuit of light and darkness. Daily visiting of the animals during the whole procedure to detect distress was done and animals that suffer of any type of distress, like fatigue or pain were euthanatized. Rats (n = 36) underwent inhalation exposure of carbon tetrachloride (CCl_4_, Sigma-Aldrich, Steinheim, Germany) three times a week (approx. 5ml CCl_4_ per animal and inhalation). Phenobarbital (0.35g/l, Sigma-Aldrich, Steinheim, Germany) was added to the drinking water as described previously [8]. Treatment was given for 8 weeks (early cirrhosis without ascites) or 12–14 weeks (advanced cirrhosis with ascites). Isolation of primary cells was performed 6–10 days after the last doses of CCl_4_ and phenobarbital. Age-matched rats were used as control group.

Additional male Wistar rats (n = 12) underwent inhalation exposure of CCl_4_ as described above. Starting at week 8 the partial Glo-I inhibitor ethyl pyruvate (EP; 40 mg/kg b.w. i.p. daily) or an equivalent amount of 0.9% saline solution i.p. instead of EP was given and treatment with CCl_4_ continued until 12–14 weeks. Rats were sacrificed 6–10 days after the last doses of CCl_4_, Phenobarbital and EP.

The American Physiological Society guide principles for the care and use of animals were followed. The Landesverwaltungsamt Sachsen-Anhalt (Institutional Animal Care and Use committee) previously approved all procedures involving animals (42502-2-1223MLU).

### Isolation of primary liver cells

Primary rat hepatocytes (pHEP), primary hepatic stellate cells (pHSC) and primary liver sinusoidal endothelial cells (pLSEC) were isolated from male Wistar rats as described previously [9–12]. Briefly, rats were anesthetized using ketamine hydrochloride (Ketavet, Pfizer, Berlin, Germany, 100mg/kg body wt) and xylazine (Rompun, Bayer, Leverkusen, Germany, 40mg/animal). After laparotomy, the portal vein and inferior vena cava were cannulated and liver was perfused with oxygenated (carbon gas, 95% O_2_, 5% CO_2_) Krebs–Henseleit solution containing dextrose (11mM, Sigma-Aldrich, Steinheim, Germany) and collagenase (0.7mg/ml, Serva, Heidelberg, Germany) for pHEP or pronase and DNase (Roche, Mannheim, Germany) for pHSC and pLSEC at 37°C for 20 min. Cannulation of portal vein led to decease of rats due to exsanguination. The liver was then excised, minced and passed through a series of nylon mesh filters (100μm, Sigma-Aldrich, Steinheim, Germany) and centrifuged at 50 x g for 5 minutes for cell isolation.

For culturing, pHEP were plated 2h with MEM + 2% FCS and 1% penicillin/streptavidin (P/S, Sigma-Aldrich, St. Louis, USA) on collagen-coated dishes and afterward with HGM-Medium including HGF+EGF (PromoCell, Heidelberg, Germany). pLSEC were seperated through a 25%/50% Percoll gradient by centrifugation (800 x g for 25 minutes at 4°C). Cells were plated on 10cm-plates (TPP, Trasadingen, Switzerland) and incubated at 37°C for 20 minutes. The nonadherent cells were cultured for 1 day in RPMI (RPMI1640, PAA, Pasching, Austria) with 10% FCS and 1% P/S. Cells were collected in trizol (Qiazol, Qiagen, Hilden, Germany) for RNA isolation or protein lysis buffer (IPP) for protein isolation. pLSEC were cultivated in RPMI, pHSC in IMDM (Gibco, NY, USA) with 10% FCS and 1% PS.

### Western blot analysis

Protein lysates were boiled for 5 min at 95°C in SDS protein buffer (Thermo-Scientific, Rockford, USA) and separated by SDS-PAGE following transfer to PVDF membrane. Primary antibodies were Glo-I (SC-67351), NF-ĸB (p65 subunit, SC-372), Nrf2 (SC-722), ERK1 (all rabbit polyclonal IgG, SC-94), pERK (mouse monoclonal IgG2a, SC-7383), Vinculin (rabbit polyclonal IgG, SC-5573, all Santa Cruz Biotechnology, Dallas, Texas, USA), α-SMA (rabbit polyclonal IgG, ab5694,), TGF-β (rabbit polyclonal IgG, ab66043, both abcam, Cambridge, UK) and actin (mouse monoclonal AB, MAB1501, Millipore, California, USA). Secondary antibodies were anti-mouse (IgG-HRP, 7076P2, horse origin), anti-rabbit (IgG-HRP, 7074P2, goat origin, all Cell Signaling Technology, Boston, Massachusetts, USA) and anti-goat (IgG-HRP, 705-035-003, donkey origin, Dianova, Hamburg, Germany). Western blot signals were quantified using imager (Fusion-Fx-7 with BD-Software, Peqlab, Erlangen, Germany). Signals were normalized to its respective loading controls using ImageJ-Software (v. 1.48, http://imagej.nih.gov).

### Measurement of Glo-I activity

Activity of glyoxalase I (Glo-I, E.C.4.4.1.5) was determined by measurement of the reaction intermediate S-D-lactoylglutathione with ascending absorbance at 240nm [13]. Absorbance was measured for 5 min at 25°C in crystal cuvette (Hellma, Berlin, Germany) at photometer (amersham ultrospec 2100 pro, amershampharmacia biotech, Cambridge, England). For each test 2mM GSH (Roth, Karlsruhe, Germany) and 2mM MGO (Sigma-Aldrich, Steinheim, Germany) were incubated for 90 sec in 50mM phosphate-buffer (Na_2_HPO_4_, pH 7.0, Roth, Karlsruhe, Germany) and 10μl of undiluted cell lysate were used per test. Each probe was measured three times. Phosphate-buffer was set as reference. Enzyme activity was calculated in U by formula: A = (ΔE/min x V) / (ε x d x v). ε for S-D-lactoylglutathione was 2.86 (mol/l x cm). For specific activity U were referred to protein-concentration.

### Inhibition of Glo-I activity in hepatic stellate cells with Ethyl Pyruvate (EP) or BrBzGSHCp_2_

Hepatic stellate cells (HSZ-B-S1) were seeded and then treated with EP (1-20mM) or BrBzGSHCp_2_ (1–10μM) and/or 100ng/ml LPS (all from Sigma-Aldrich, Steinheim, Germany) in serum-free medium. EP and BrBzGSHCp_2_ are two different inhibitors of Glo-I [14,15]. In order to determine Glo-I activity, cells were washed twice after 24h incubation, lysed and centrifuged at 13000 x g and 4°C for 15 min. Supernatants were collected and stored at -20°C. Protein concentrations were determined using BCA-method following instructions of the manufacturer (Sigma-Aldrich, Steinheim, Germany). For evaluation of the effect of partial inhibition of Glo-I activity, supernatants were collected and frozen at -20°C. For TNF-α-ELISA (MyBiosource, San Diego, USA) 200μl supernatant, for collagen-I-ELISA (MyBiosource, San Diego, USA) 40μl supernatant and for α-SMA-ELISA (MyBiosource, San Diego, USA) 40μl supernatant were used following instructions of the manufacturer.

### Partial inhibition of Glo-I activity with Ethyl Pyruvate (EP) during progression of cirrhosis

Rats were treated with EP or saline as described above. Liver samples were fixed using 4% formaldehyde, after embedding in paraffin, 4-μm sections were stained with sirius red as described before [16]. Overview pictures and liver sections were analyzed using the Keyence Biozero BZ 8000 microscope with BZ Viewer (Osaka, Japan). At least 4 livers for each group were analyzed with 10 sections per liver from an investigator blinded for the different treatment groups. Area of sirius red was analyzed with ImageJ and MRI_Fibrosis_Tool-Plugin (http://dev.mri.cnrs.fr/projects/imagej-macros/wiki/Fibrosis_Tool).

### Statistics

Results are expressed as mean ±SD. Comparisons between groups were analyzed by one-way ANOVA followed by post-hoc Bonferroni correction to detect differences between groups. P values <0.05 were considered as statistically significant. GraphPad Prism 4.0 software was used.

For additional description see S1 File.

## Results

### Expression and specific activity of Glo-I in normal primary liver cells

**Fig 1:**
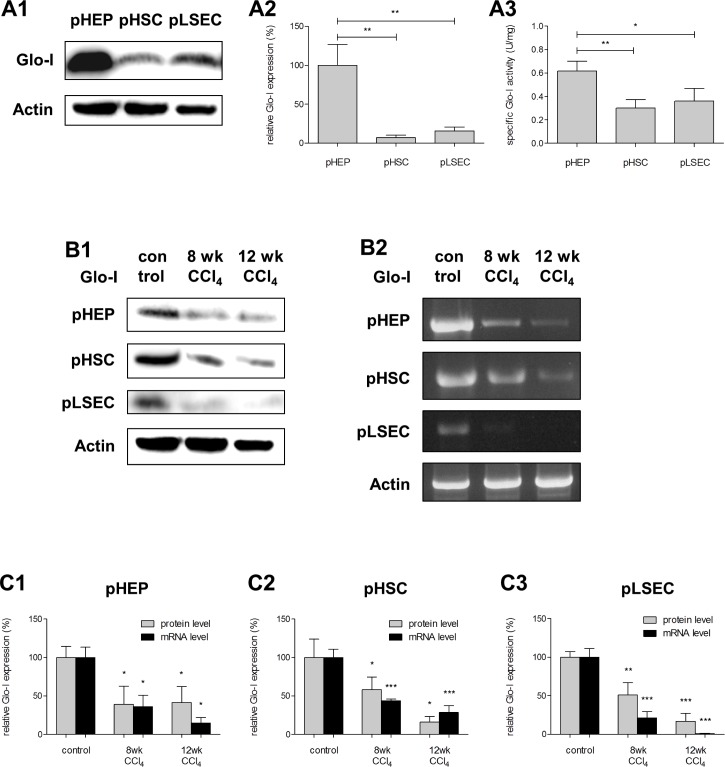
Protein and mRNA expression of Glo-I in pHEP, pHSC and pLSEC in controls, early and advanced cirrhosis. **A1**, Protein analysis by Western blot of Glo-I in pHEP, pHSC and pLSEC indicating highest expression in pHEP. Quantification **(A2)** of three independent experiments showed significant lower Glo-I expression in pHSC and pLSEC. **A3**, Specific activity of Glo-I in pHEP, pHSC and pLSEC. Enzyme kinetics showed highest specific activity of Glo-I in pHEP (0.62 U/mg). Lower specific Glo-I activity was found in pHSC (0.30 U/mg) and pLSEC (0.36 U/mg). **B1-B2**, Reduction of Glo-I in early (8 wk CCl_4_-treatment) and advanced cirrhosis (12 wk CCl_4_-treatment) showed by Western blot (**(A1)**, PROT) and RT-PCR (**(A2)**, RNA) in primary hepatocytes (pHEP), primary hepatic stellate cells (pHSC) and primary liver sinusoidal endothelial cells (pLSEC). Quantification **(C1-C3)** of at least three independent experiments confirmed significantly reduced expression of Glo-I in early cirrhosis (8 wk of CCl_4_-treatment) in isolated pHEP (PROT: 39.1±13.7%, p = 0.018; RNA: 25.0±15.0%, p = 0.030), pHSC (PROT: 58.2±9.5%, p = 0.041; RNA: 43.4±1.4%, p<0.001) and pLSEC (PROT: 51.2±9.0%, p = 0.008; RNA: 21.5±4.5%, p<0.001) compared to normal (100%). In advanced cirrhosis (12 wk of CCl_4_-treatment) expression of Glo-I was significantly reduced in protein and mRNA levels in pHEP (PROT: 41.5±12.0%, p = 0.016; RNA: 9.9±13.5%, p = 0.014), pHSC (PROT: 15.9±4.3%, p = 0.043; RNA: 28.6±4.9%, p<0.001) and pLSEC (PROT: 16.5±5.9%, p<0.001; RNA: 0.7±0.3%, p<0.001) compared to normal (100%). Results are expressed as mean ± S.D. * P<0.05, ** P<0.01, *** P<0.001.

In control animals both protein and mRNA expression of Glo-I was detected in all explored cells. Taking the expression in hepatocytes as the reference (pHEP; PROT: 100.0±15.3%; RNA: 100.0±7.6%), a relatively lower expression of Glo-I was found in primary hepatic stellate cells (pHSC; PROT: 7.0±1.9%, p = 0.038; RNA: 6.2±0.8%, p = 0.003) and primary sinusoidal endothelial cells (pLSEC; PROT: 16.1±2.7%, p = 0.057; RNA: 10.8±3.0%, p<0.001, Fig 1A1 and 1A2). Despite the lower expression of Glo-I in the latter, the specific activity of the enzyme was proportionally only slightly higher in pHEP (pHEP: 0.62±0.04 U/mg; pHSC 0.30±0.03 U/mg, p = 0.001; pLSEC: 0.36±0.06 U/mg, p = 0.016, Fig 1A3).

### Expression and specific activity of Glo-I in cirrhosis

**Fig 2:**
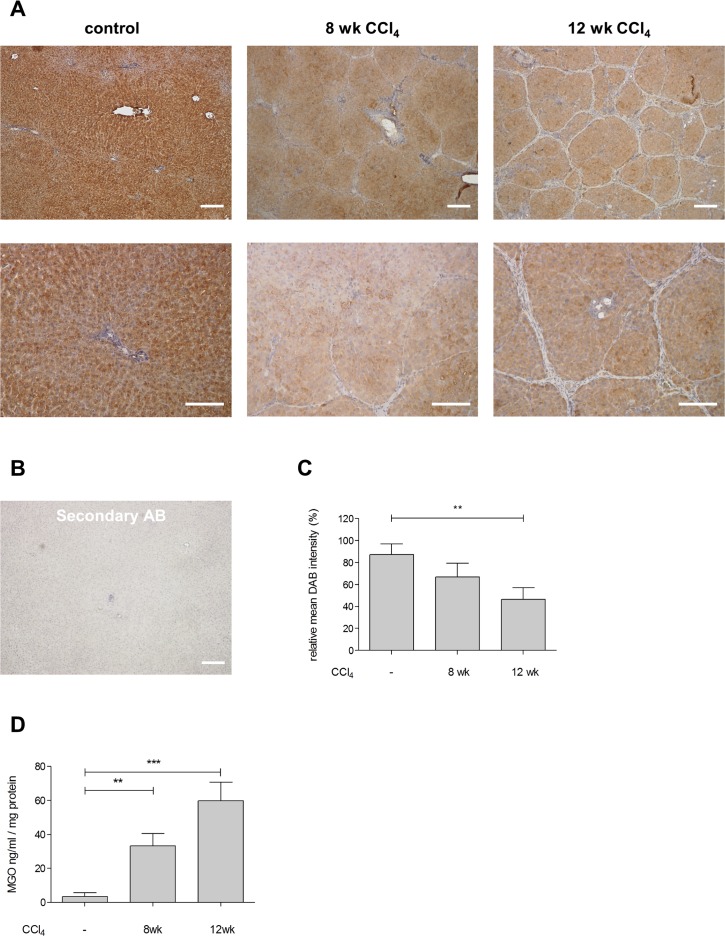
Expression of Glo-I and MGO in whole rat liver. **A**, Sections of Wistar rat liver with DAB-staining showing controls, early cirrhosis after 8 wk CCl_4_-treatment and advanced cirrhosis after 12 wk CCl_4_-treatment at 5x (upper line) or 20x (lower line) magnification. Rats underwent inhalation exposure of CCl_4_ three times a week, Phenobarbital was added to the drinking water. Isolation of livers was performed 6–10 days after the last doses of CCl_4_ and phenobarbital. Age-matched rats were used as control group. **B**, Control section lacking the primary Glo-I antibody revealed no staining. **C**, Quantification of at least 3 livers with every 10 sections showed significant reduction in staining intensity and Glo-I expression in early and advanced cirrhosis **D**, Analysis of MGO in controls, early and advanced cirrhosis via ELISA. Quantification of at least three independent experiments revealed significantly elevated MGO levels in cirrhosis after 8 wk and 12 wk CCl_4_ treatment. Scale bars: 100μm (A, upper line), 50μm (A, lower line). * P<0.05, ** P<0.01, *** P<0.001.

**Fig 3:**
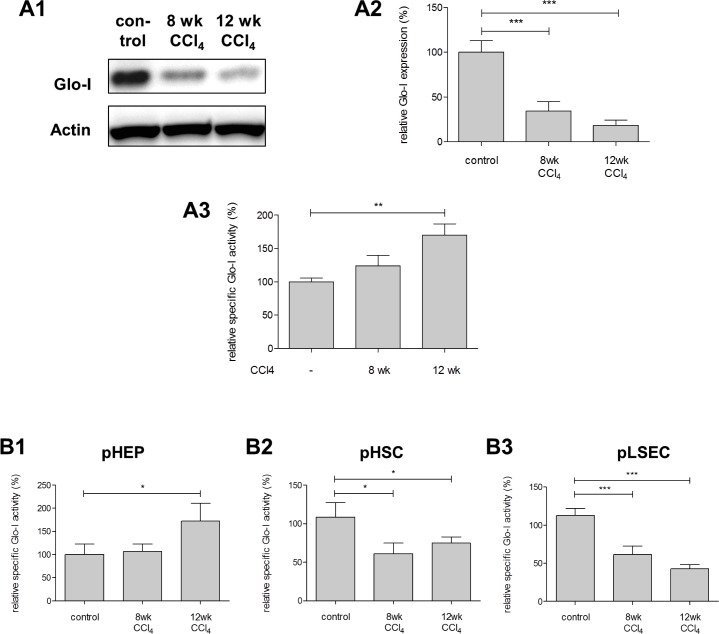
Protein expression and specific activity of Glo-I in whole liver and specific activity of Glo-I in pHEP, pHSC and pLSEC in controls, early and advanced cirrhosis. **A1**, protein analysis of Glo-I by Western blot in Wistar rat livers indicating reduced protein expression after 8 and 12 wk CCl_4_-treatment. Quantification **(A2)** of three independent experiments showed significant reduction of Glo-I in cirrhosis compared to controls. **A3**, relative specific Glo-I activity was increased in early and advanced cirrhosis. Average specific Glo-I activity in controls was 1.074 U/mg. Primary liver cells were isolated in controls and livers with early or advanced cirrhosis using portal vein perfusion with pronase and DNAse. Specific Glo-I-activity was reduced in cirrhosis in primary hepatic stellate cells (pHSC **(B2)**) and primary liver sinusoidal endothelial cells (pLSEC **(B3)**) but elevated in primary hepatocytes (pHEP **(B1)**). Results are expressed as mean ± S.D. * P<0.05, ** P<0.01, *** P<0.001.

Expression of Glo-I was reduced in early and advanced cirrhosis in whole liver compared to controls in both immunohistochemistry (IHC, Fig 2) and Western blot analysis (Fig 3A1 and 3A2). Furthermore, animals with advanced cirrhosis had a greater reduction in Glo-I expression (12 wk CCl_4_: IHC 46.6±4.7%, WB 18.4±2.1%) than early cirrhosis (8 wk CCl_4_: IHC 66.9±6.2%; WB 34.3±4.2%;) compared to controls (100%, p = 0.017 and p<0.001 for IHC and WB, respectively, Figs 2 and 3).

Similar results were also observed in primary cells. In all liver cirrhosis cells, and compared to controls, protein and mRNA levels of Glo-I were significantly decreased both in early and advanced cirrhosis (Fig 1B1–1C3). Furthermore, a stepwise decrease in Glo-I expression was observed in pHSC (8 wk vs. 12 wk CCl_4_: 58.2±9.4% vs. 15.9±4.3%, p = 0.015) and pLSEC (8 wk vs. 12 wk CCl_4_: 51.2±9.0% vs. 16.8±5.9%, p = 0.033) with increasing severity of disease but not in pHEP (8 wk vs. 12 wk CCl_4_: 39.1±13.7% vs. 41.5±12.0%, p = 0.90).

When analyzing cells of liver cirrhosis, the specific Glo-I activity was reduced in pHSC (8 wk CCl_4_: 60.0±8.0%, p = 0.025; 12 wk CCl_4_: 74.8±4.7%, p = 0.048) and pLSEC (8 wk CCl_4_: 61.5±6.3%, p = 0.004; 12 wk CCl_4_: 42.9±3.1%, p<0.001; Fig 3B2 and 3B3) in cirrhosis compared to controls (100%). Nevertheless, in pHEP an increase in the activity was observed in advanced cirrhosis (12 wk CCl_4_: 172.0±22.3%, Fig 3B1) compared to controls (100%, p = 0.049); in these cells no difference was observed when comparing early cirrhosis to controls (8 wk: 107.0±9.3%, p = 0.68, Fig 3B1). Like in pHEP, analysis of whole liver lysates showed elevated specific Glo-I activity in cirrhosis compared to control (12 wk CCl_4_: 170±16.7% vs. 100±5.6%, p = 0.0023, Fig 3A3).

### MGO in cirrhosis

In order to further analyze the role of Glo-I in cirrhosis, we measured MGO levels in normal and cirrhotic livers. The results revealed a significant increase of MGO concentrations in cirrhosis. MGO levels were significantly elevated after 8 wk of CCl_4_ treatment (33.2±7.4 ng/ml compared to 3.4±2.3 ng/ml in controls, p = 0.003), and even higher after 12 wk of CCl_4_ (59.7±3.4 ng/ml; vs control p<0.001). The increase of MGO concentrations from week 8 to 12 also reached significance (p = 0.03).

### Effect of partial inhibition of Glo-I in HSC on the secretion of proinflammatory cytokines and markers of fibrosis

The expression and activity of Glo-I were confirmed in hepatic stellate cell line (data not shown). Partial inhibition of specific Glo-I activity by EP was demonstrated without effect on Glo-I expression (S2A1 and S2A2 Fig). We found dose dependent significant inhibition of Glo-I enzyme activity in HSC (S2B Fig), that reached significance using a dose of 10mM EP (73.0±1.7%, p = 0.002). Doses of 15mM and more were prior shown to inhibit Glo-I activity [14]. We could confirm these results in HSC by using 1-10mM of EP. The significant inhibition of Glo-I activity was not detectable using 20 mM of EP (S2B Fig).

Glo-I activity of hepatic stellate cells after inflammatory stimulus was evaluated (S2C Fig). Administration of LPS led to a significant increase of Glo-I activity in HSC after incubation for 24h. Treatment with EP alone or co-incubation of LPS and EP resulted in significant reduction of specific Glo-I activity confirming partial inhibition of the enzyme. Furthermore, EP resulted in significant increase of MGO levels after 24h incubation in HSC (1.03±0.2 vs. 0.34±0.1 ng/ml, p = 0.004, S2D Fig).

**Fig 4:**
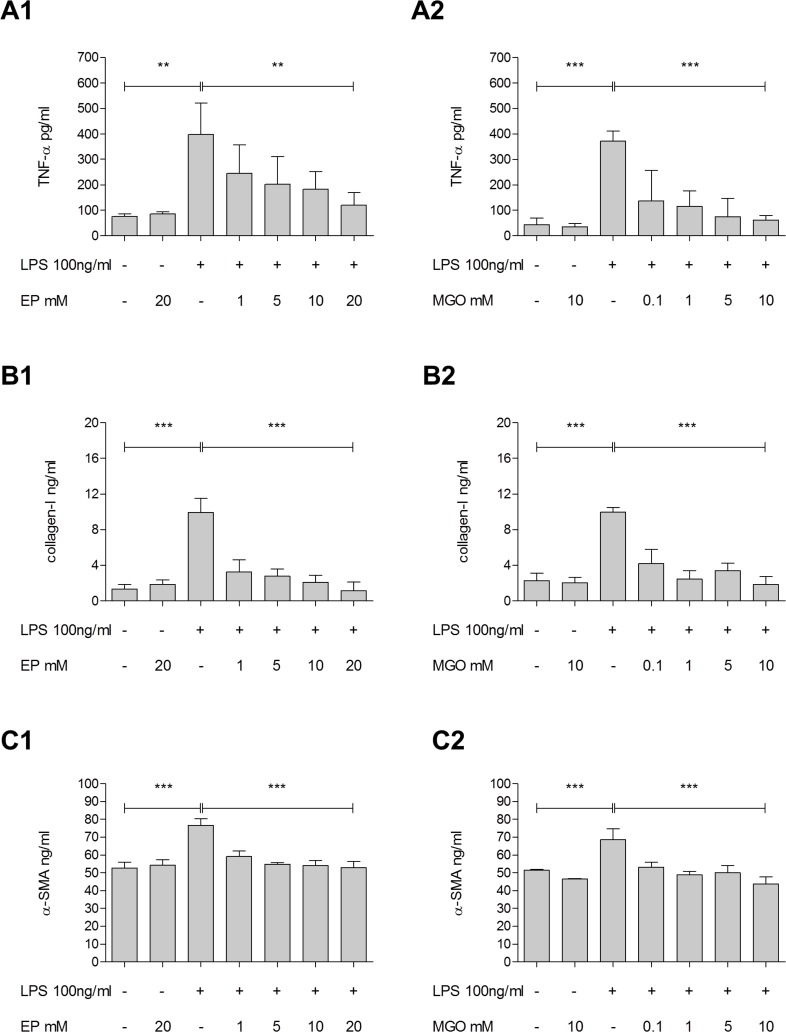
Effect of partial Glo-I inhibition by EP on LPS-induced production of TNF-α, collagen-I and α-SMA in HSC. **A1-C2**, HSC were incubated for 24h with 100ng/ml LPS and 1-20mM EP **(A1, B1, C1)** or 0.1-10mM MGO **(A2, B2, C2)**. Supernatants were analyzed via ELISA for TNF-α, collagen-I and α-SMA. LPS stimulation led to significantly increased levels of TNF-α, collagen-I and α-SMA. Co-treatment of LPS and EP indicated significant dose-dependent reduction of LPS-induced markers of inflammation **(A1)** and fibrosis **(B1, C1)**. Presence of LPS and treatment with low-level concentrations of MGO (indicating partial inhibition of Glo-I activity without complete enzyme inhibition) showed significant decrease of LPS-induced release of TNF-α (absence vs. presence of 10mM MGO: 371.8±22.7 vs. 60.9±11.2 pg/ml; p<0.001), collagen-I (10.0±0.3 vs. 1.8±0.5 ng/ml; p<0.001) and α-SMA (68.6±3.6 vs. 43.8±2.2 ng/ml; p<0.001) **(A2, B2, C2)**. Presence of EP or MGO in absence of LPS revealed no changes in the concentration of TNF-α, collagen-I and α-SMA. Results are expressed as mean ± S.D. of at least three independent experiments. * P<0.05, ** P<0.01, *** P<0.001.

Treatment with EP led to a significant dose-dependent reduction of LPS-induced release of TNF-α (absence vs. presence of 20mM EP: 397.1±71.3 vs. 121.0±28.2 pg/ml; p = 0.06), collagen-I (9.9±0.9 vs. 1.2±0.5 ng/ml; p<0.001) and α-SMA (76.7±2.0 vs. 52.9±2.0 ng/ml; p<0.001) in hepatic stellate cell line (Fig 4). Doses of 10mM (data not shown) or 20mM (Fig 4) of EP without LPS stimulation showed no effects in expression of TNF-α, collagen-I or α-SMA.

In order to evaluate whether these effects are due to the action of EP on Glo-I, similar experiments with LPS and low-level concentrations of MGO (with simulates the effect of EP on Glo-I) were performed. Again, a significant decrease of LPS-induced release of TNF-α, collagen-I and α-SMA were observed. In the absence of LPS, neither the administration of EP or MGO led to changes in the concentration of these aforementioned cytokines.

**Fig 5:**
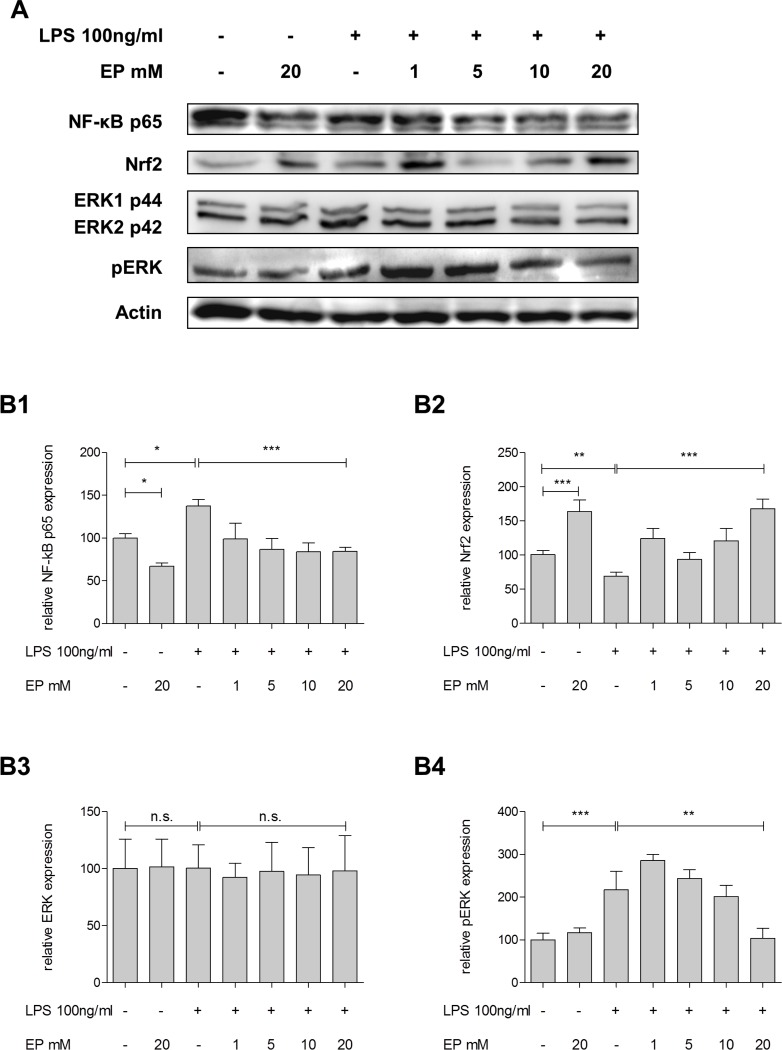
Effect of partial Glo-I inhibition by EP to NF-kB, Nrf2 and ERK-pathways. **A-B4**, HSC were incubated for 24h with 100ng/ml LPS and 1-20mM EP. Protein analysis by Western blot indicated significant reduction of NF-kB p65 subunit by EP and attenuation of LPS-induced NF-kB stimulation **(A, B1)**. EP led to significant elevation of Nrf2 and EP diminished LPS-induced reduction of Nrf2 **(A, B2)**. EP treatment significantly dimished LPS-stimulated elevation of pERK **(A, B4)** without significant change in ERK **(A, B3)**. Results are expressed as mean ± S.D. of at least three independent experiments. * P<0.05, ** P<0.01, *** P<0.001.

Similarly, treatment with EP showed significant decrease of LPS-induced NF-kB stimulation in a dose-dependent manner (absence vs. presence of EP 20mM: 137.5±4.4% vs. 84.5±2.6%; p<0.001; Fig 5A and 5B1). Treatment with EP inhibited the LPS-induced reduction of Nrf2 (69.2±3.3% vs. 168.1±8.1%; p<0.001, Fig 5A and 5B2). Furthermore, increasing concentrations of EP reduced LPS-induced pERK (217.3±25.1% vs. 103.9±13.5%; p = 0.008; Fig 5A and 5B4) without effect on the ERK expression (100.0±14.9% vs. 97.9±17.9%; p = 0.933; Fig 5A and 5B4).

### Effect of Glo-I inhibitor BrBzGSHCp_2_ on markers of inflammation and fibrosis in HSC

In order to confirm our results we used an additional Glo-I inhibitor, S-p-bromobenzylglutathione cyclopentyl diester [15] (BrBzGSHCp_2_). BrBzGSHCp_2_ showed significant dose dependent inhibition of specific Glo-I activity with significant reduction at doses of 5μM (p = 0.01) and 10μM (p = 0.02, S3A Fig). Furthermore, treatment with BrBzGSHCp_2_ resulted in significantly reduced expression of α-SMA (10μM vs. control: 20±3% vs. 100±13%, p<0.001), TGF-β (18±11% vs. 100±15%, p = 0.002) and NF-κB (4±2% vs. 100±9%, p<0.001) with stimulation of Nrf2 (237±10% vs. 100±12%, p<0.001).

### Effect of partial inhibition of Glo-I on progression of cirrhosis

**Fig 6:**
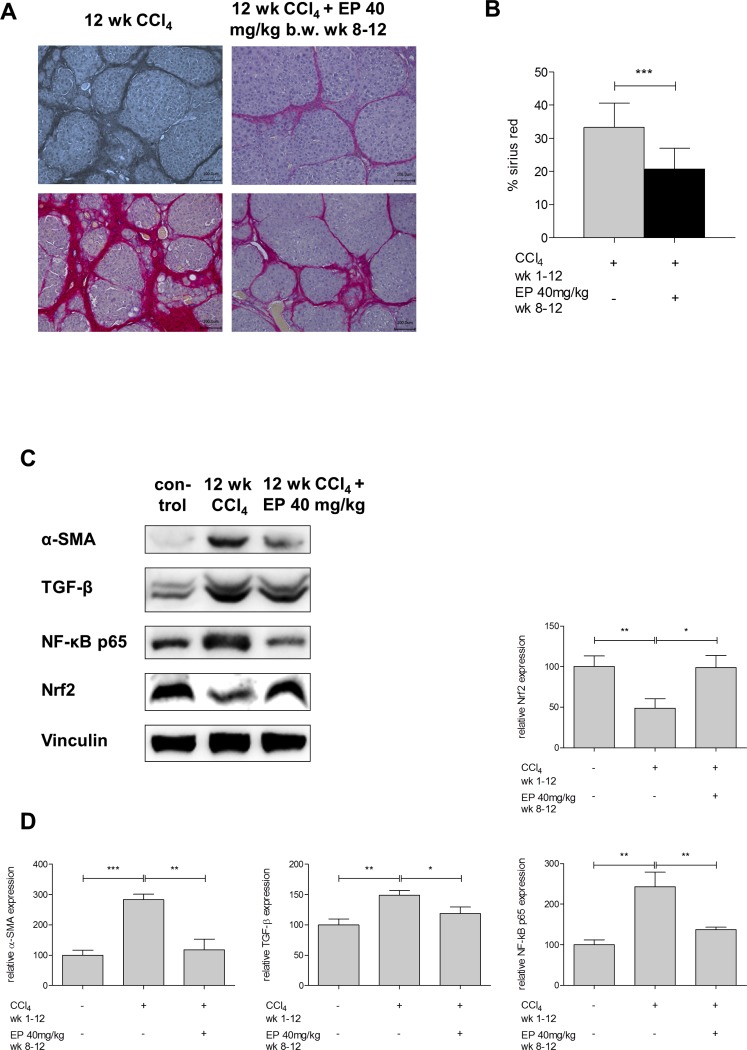
Effect of partial Glo-I inhibition by EP on progression of cirrhosis and markers of inflammation and fibrosis. Sections of Wistar rat livers with sirius red staining showed advanced cirrhosis after 12 wk CCl_4_-treatment and treatment with or without EP at 20x magnification **(A)**. EP-group received 40 mg/kg b.w. EP i.p. once daily from week 8–12, control group received 0.9% NaCl i.p.. Two representative Images were shown for each group. Quantification of at least 4 livers with 10 sections each per group **(B)** showed significant reduction in sirius red positive area in EP-treatment group. **C**, Markers of inflammation and fibrosis in cirrhotic livers and effect of partial inhibition of Glo-I by EP. After 12 wk CCl_4_ treatment isolated livers showed significantly elevated protein levels of α-SMA, TGF-β, NF-κB p65 and reduced expression of Nrf2. Quantification **(D)** of three independent experiments showed abolished expression alteration in EP group in markers of inflammation and fibrosis. Scale bars: 100μm. Results are expressed as mean ± S.D. * P<0.05, ** P<0.01, *** P<0.001.

In order to evaluate the effect of partial inhibition of Glo-I on development and progression of cirrhosis we administered EP i.p. to Wistar rats, which underwent CCl_4_-inhalation for induction of cirrhosis. The treatment group received additional EP from week 8–12 i.p. daily while in the control group saline was given. After 12 weeks of CCl_4_ administration the amount of fibrotic tissue in the control group was 33.3±7.3% whereas the EP-treated group revealed significant lower sirius red content (20.7±6.2%; p<0.001; Fig 6A and 6B).

For further analysis we determined expression of α-SMA, TGF-β, NF-κB and Nrf2 in cirrhotic livers and evaluated effect of *in vivo* EP treatment. Compared to controls, cirrhotic livers showed significantly higher expression of α-SMA (284±18% vs. 100±17%, p<0.001), TGF-β (148±8% vs. 100±10%, p = 0.003) and NF-κB (243±36% vs. 100±12%, p = 0.003) with reduced expression of Nrf2 (49±12% vs. 100±13%, p = 0.008). Treatment with EP significantly reduced the expression of α-SMA (118±35%, p = 0.002), TGF-β (119±11%, p = 0.02), NF-κB (137±6%, p = 0.008) and raised levels of Nrf2 (99±15%, p = 0.01, Fig 6C and 6D) compared to cirrhotic livers without EP treatment.

## Discussion

Our study aimed at analyzing the expression and activity of Glo-I in cirrhosis and evaluating the effect of partial Glo-I inhibition in hepatic stellate cells and on progression of cirrhosis. We observed a significant reduction of Glo-I in cirrhosis compared to controls, both on protein and mRNA levels accompanied by elevated levels of MGO in cirrhosis. Furthermore, the reduction in Glo-I expression as well as the increase in MGO concentration were greater with increasing severity of liver disease. This reduction in cirrhosis was found in the whole liver as well in all explored liver cells, namely hepatocytes, hepatic stellate cells and sinusoidal endothelial cells. In addition we observed a decreased activity of Glo-I in hepatic stellate cells. On the other hand, stimulation of non-cirrhotic HSC with LPS as would occur hypothetically in an initial stadium of liver disease, resulted in significant increase of specific Glo-I activity. Finally, we could clearly show that reduction of Glo-I activity with two different inhibitors led to a reduced activation of hepatic stellate cells as shown by a decrease in the secretion of TNF-α, collagen-I and α-SMA. *In vivo* experiments confirmed the antifibrotic properties of inhibition of Glo-I. Partial inhibition of Glo-I by EP resulted in significantly reduced amount of fibrotic tissue and reduced markers of inflammation and fibrosis. Interestingly, this effect was present starting the administration of EP in already established cirrhosis.

Glo-I is the main enzyme responsible for the detoxification of MGO, which is cytotoxic in high concentrations via formation of AGEs. AGEs bind to their receptor RAGE and stimulates different signaling pathways involved in inflammation that lead to activation of hepatic stellate cells [17]. According to our results, we hypothesize that the reduction in the expression and activity of Glo-I in the hepatic stellate cells in cirrhosis perpetuates liver injury as demonstrated by elevation of MGO levels. Indeed, previous studies have described similar findings in other chronic inflammatory diseases such as atherosclerosis, in which a decrease in Glo-I expression and increase in MGO is observed in atherosclerotic plaques as a consequence of the chronic inflammation [18]. In this regard it has been demonstrated that Glo-I expression is elevated upon acute oxidative stress whereas formation of subchronic oxidative stress leads to reduced expression of Glo-I [19]. Our results observing a greater reduction of Glo-I with increasing severity of liver disease (8 weeks vs. 12 weeks CCl_4_-treatment), which in turn lead to further decrease of Glo-I, suggest that there is a “vicious circle” which propagate itself in liver disease.

Several results from this study may seem somewhat counterintuitive. Firstly, the effect of the partial inhibition of Glo-I activity with EP leading to a reduction in the production of fibrosis markers in normal hepatic stellate cell cultures is difficult to understand given the decreased activity (and expression) of Glo-I in hepatic stellate cells observed in cirrhosis. In this regard our results revealed in non-cirrhotic HSC an increase of Glo-I activity upon LPS stimulation, which was abrogated with partial inhibition of Glo-I by EP. Furthermore, the reduction of the activity of Glo-I, which is observed in cirrhosis, is much greater than the partial inhibition that is induced with the administration of EP. To clarify this issue we performed measurements of MGO concentrations expecting higher levels of MGO in cirrhosis. Indeed the reduction in Glo-I expression seen in cirrhosis lead to a higher concentration of MGO than with pharmacological inhibition of Glo-I, in which only a slight increase of MGO is observed. Our findings are further supported through prior analysis regarding Glo-I inhibition and MGO [15,20,21]. The fact that administration of EP *in vivo* decreased the amount of fibrosis and the progression of the disease as well as the CCl_4_-induced expression of α-SMA, TGF-β and NF-κB further affirm these theories.

Although MGO is toxic at high concentrations, a low level of MGO has been shown to activate transcription factors [22] and modify proteins [23]. In this regard, different MGO levels could lead to temporary activation or inhibitions of biochemical targets that are regulated via Glo-I activity. Interestingly, we observed that incubation of HSC with low doses of MGO in millimolar range resulted in comparable effects on LPS-induced TNF-α, collagen-I and α-SMA concentration as treatment with EP did. Indeed, previous studies suggest that MGO could act as an intracellular mediator of the action of Glo-I inhibitors, which is in line with our results [15]. Recent work showing an inhibitory effect of MGO on NF-kB [24] and transcriptional control of Glo-I by Nrf2 in response to MGO [25,26], which further supports our findings of Nrf2 stimulation via partial Glo-I inhibition by EP in cell culture and *in vivo*. These complex and dose dependent regulatory processes could also explain, that EP cause dose dependent partial inhibition of Glo-I in lower concentrations but higher concentrations could not inhibit the enzymatic Glo-I activity.

Another potential confusing finding is the observation of higher specific Glo-I activity in hepatocytes and subsequently whole liver in cirrhosis in contrast to stellate cells and endothelial cells. These results might be a consequence of cell death and repair mechanisms and are a reflection of compensatory regulations since hepatocytes are the main target of liver injury [27,28].

Our study has some limitations. Glo-I is an ubiquitous enzyme, total gene-knock-out is embryological lethal, so these animals are not available. In addition we could not clearly differentiate if the reduction of Glo-I in cirrhotic stellate cells is a cause or a consequence of cirrhosis. Our result in LPS-stimulated HSC leading to the interpretation that it is more a cause of cirrhosis. Furthermore we could not exclude, that the shown effects of EP are mediated via alternative regulatory pathways than Glo-I. To overcome these limitations, we performed *in vivo* experiments with CCl_4_-model in Wistar rats. Partial inhibition of Glo-I by EP led to significantly reduced amount of fibrotic tissue and proinflammatory as well as fibrotic markers after 12 weeks of CCl_4_-inhalation. Furthermore, we used another well-known Glo-I inhibitor, BrBzGSHCp_2_ [15]. Treatment of HSC with BrBzGSHCp_2_ resulted in similar effects regarding reduced expression of α-SMA, TGF-β and NF-κB with stimulation of Nrf2.

In conclusion we showed the importance of Glo-I as a major detoxifying enzyme for MGO in cirrhosis. The reduced expression of Glo-I in cirrhosis demonstrates a possible explanation for increased inflammatory injury and suggests a “vicious circle” in liver disease. The regulation of specific Glo-I activity as indicated by EP could be therefore an interesting new target in the prevention of fibrosis and cirrhosis.

## Supporting information

S1 Fig Glyoxalase system. Glyoxalase I and glyoxalase II comprise the glyoxalase system for detoxification of MGO [7]. Glutathione is necessary as cofactor and is regenerated by Glo-II.
(TIFF)

S2 Fig Effect of EP and LPS on Glo-I expression, specific activity and MGO. **A1-A2**, Western blot analysis of 24h EP-treatment in doses of 1-20mM indicated no effect in Glo-I expression in HSC cell line **(A1)**. Quantification **(A2)** of at least three independent experiments showed no significant alteration in Glo-I expression. **B**, 24h EP-treatment led to concentration-dependent partial inhibition of specific Glo-I activity in doses between 1 and 10mM in HSC cell line. Doses of 20mM showed no significant enzyme inhibition. Statistically significant reduction of Glo-I was found at 10mM doses (100±3.9% vs. 73.0±1.7%, p = 0.002). **C**, HSC were incubated for 24h in presence or absence of 100ng/ml LPS and/or 10mM EP. Treatment with EP led to significant partial inhibition of Glo-I (100±6.3% vs. 69.3±8%, p = 0.04). Stimulation of HSC with LPS resulted in elevation of Glo-I activity (226.3±42%, p = 0.04). Coincubation with LPS and EP abrogated LPS-induced stimulation of Glo-I activity (75.2±11.7%, p = 0.03). **D**, Effect of EP on MGO levels. 24h treatment of HSC with 10mM EP resulted in significantly elevated MGO levels measured via ELISA. Results are expressed as mean ± S.D. of at least three independent experiments. * P<0.05, ** P<0.01, *** P<0.001.
(TIFF)

S3 Fig Effect of Glo-I inhibitor BrBzGSHCp_2_ on HSC. Effect of Glo-I inhibition on markers of inflammation and fibrosis by Glo-I inhibitor S-p-bromobenzylglutathione cyclopentyl diester (BrBzGSHCp_2_). **A**, BrBzGSHCp_2_ revealed dose dependent significant inhibition of specific Glo-I activity after 24h treatment of HSC. **B**, Western blot analysis of α-SMA, TGF-β, NF-κB p65 and Nrf2 in HSC. Quantification **(C)** showed dose dependent significantly reduced expression of α-SMA, TGF-β and NF-κB after 24h treatment with BrBzGSHCp_2_ and significant stimulation of Nrf2. Results are expressed as mean ± S.D. of at least three independent experiments. * P<0.05, ** P<0.01, *** P<0.001.
(TIFF)

S1 File Supplemental material and methods. (DOCX)

## Abbreviations

AGEs: advanced-glycation-endproducts
BrBzGSHCp_2_: S-p-bromobenzylglutathione cyclopentyl diester
CCl_4_: carbon tetrachloride
EP: ethyl pyruvate
FCS: fetal calf serum
Glo-I: glyoxalase I
GSH: L-glutathion
HSC: hepatic stellate cells
MGO: methylglyoxal
NF-ĸB: nuclear factor kB
Nrf2: nuclear factor (erythroid-derived 2)-like 2
pHEP: primary rat hepatocytes
pHSC: primary hepatic stellate cells
pLSEC: primary liver sinusoidal endothelial cells
PROT: protein level
RAGE: receptor for advanced glycation endproducts
RNA: mRNA level
ROS: reactive oxygen species
α-SMA: alpha-smooth-muscle-antigen
TNF-α: tumor necrosis factor α
