# Supplementary figures and images for: Expression of glyoxalase-I is reduced in cirrhotic livers: A possible mechanism in the development of cirrhosis
Source: PLoS One. 2017 Feb 23;12(2):e0171260. doi: 10.1371/journal.pone.0171260 (PMC5322979; doi:10.1371/journal.pone.0171260)

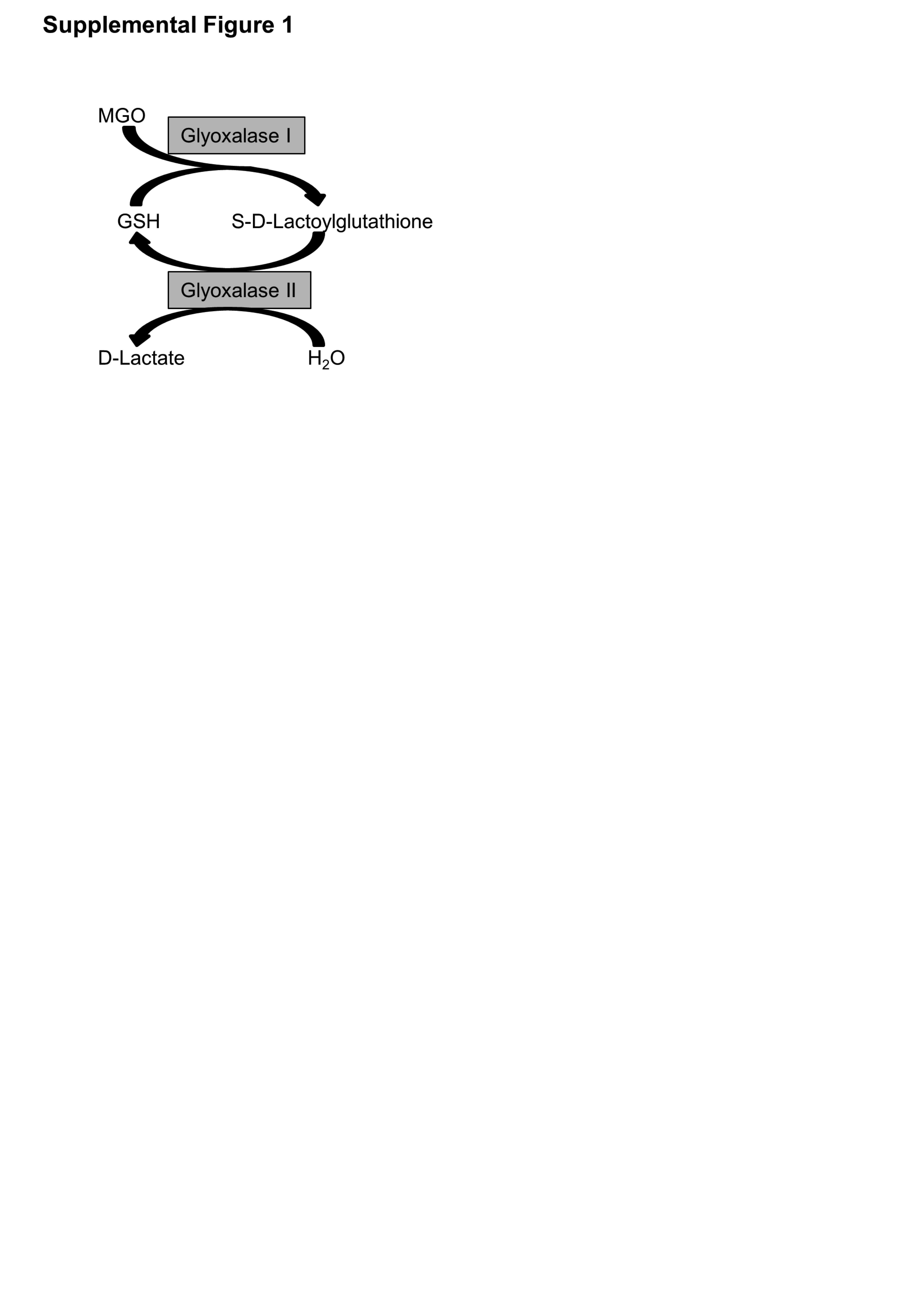

Supplement: S1 Fig — Glyoxalase I and glyoxalase II comprise the glyoxalase system for detoxification of MGO [7]. Glutathione is necessary as cofactor and is regenerated by Glo-II. (TIFF) [file pone.0171260.s001.tiff]

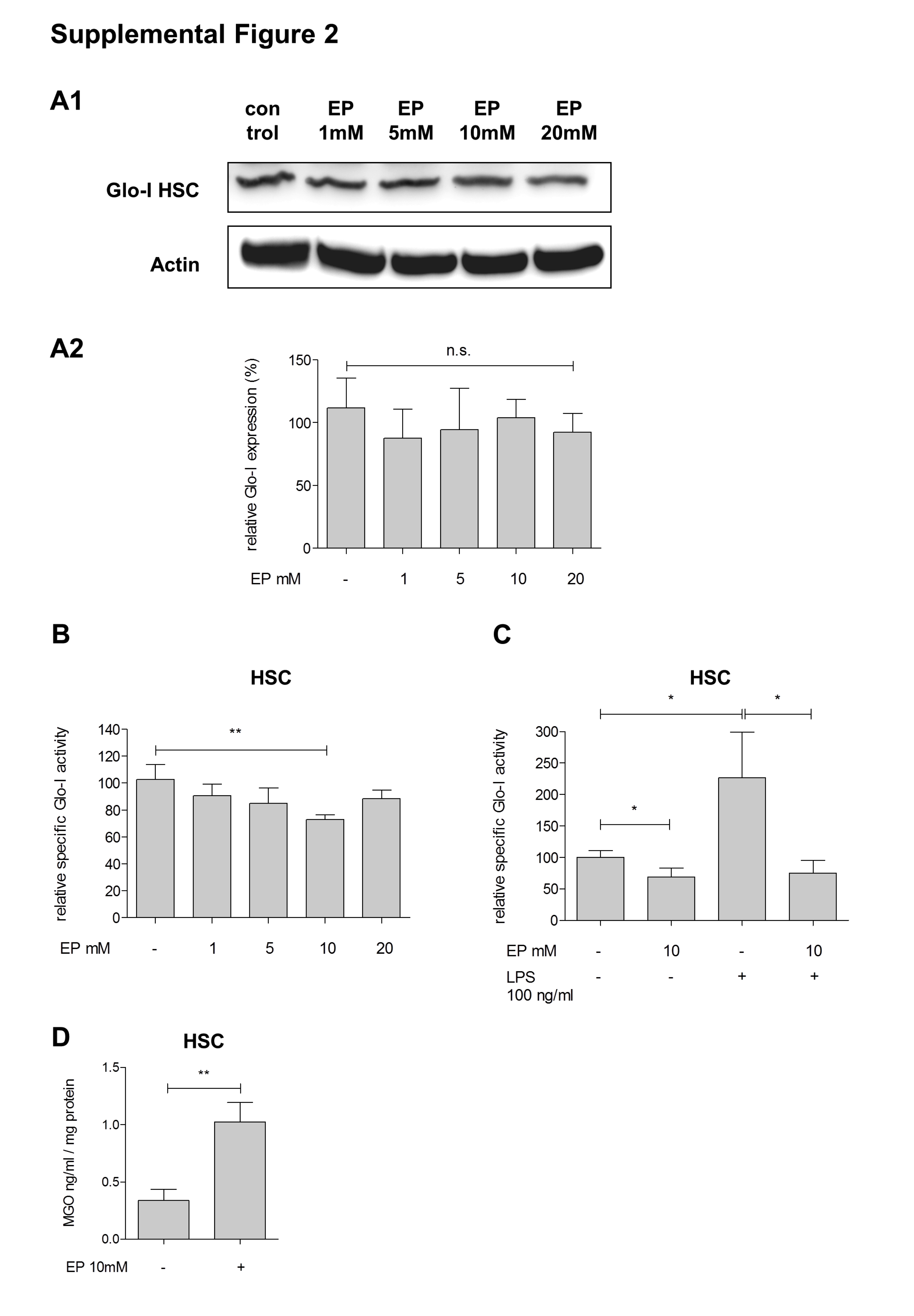

Supplement: S2 Fig — A1-A2, Western blot analysis of 24h EP-treatment in doses of 1-20mM indicated no effect in Glo-I expression in HSC cell line (A1). Quantification (A2) of at least three independent experiments showed no significant alteration in Glo-I expression. B, 24h EP-treatment led to concentration-dependent partial inhibition of specific Glo-I activity in doses between 1 and 10mM in HSC cell line. Doses of 20mM showed no significant enzyme inhibition. Statistically significant reduction of Glo-I was found at 10mM doses (100±3.9% vs. 73.0±1.7%, p = 0.002). C, HSC were incubated for 24h in presence or absence of 100ng/ml LPS and/or 10mM EP. Treatment with EP led to significant partial inhibition of Glo-I (100±6.3% vs. 69.3±8%, p = 0.04). Stimulation of HSC with LPS resulted in elevation of Glo-I activity (226.3±42%, p = 0.04). Coincubation with LPS and EP abrogated LPS-induced stimulation of Glo-I activity (75.2±11.7%, p = 0.03). D, Effect of EP on MGO levels. 24h treatment of HSC with 10mM EP resulted in significantly elevated MGO levels measured via ELISA. Results are expressed as mean ± S.D. of at least three independent experiments. * P<0.05, ** P<0.01, *** P<0.001. (TIFF) [file pone.0171260.s002.tiff]

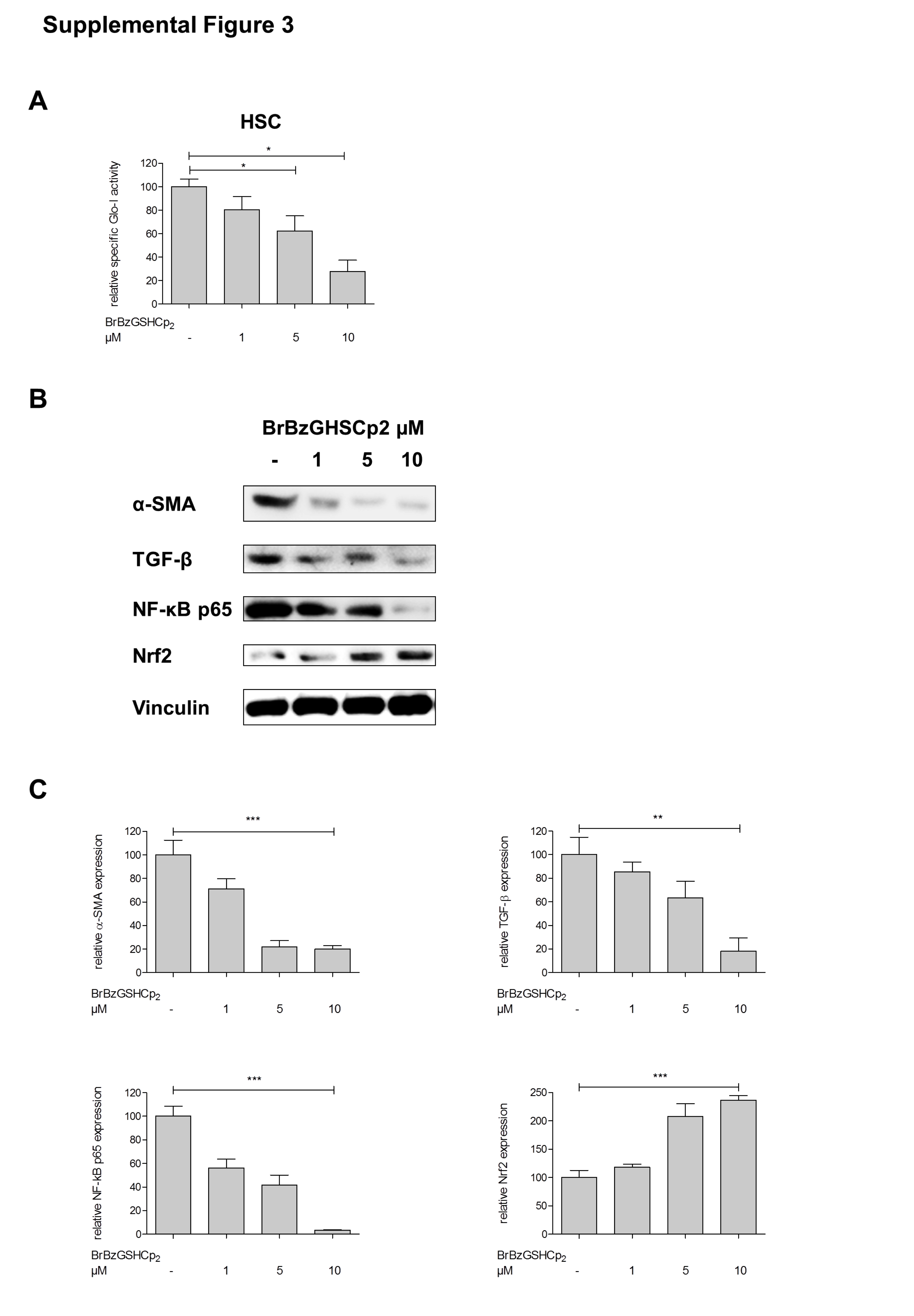

Supplement: S3 Fig — Effect of Glo-I inhibition on markers of inflammation and fibrosis by Glo-I inhibitor S-p-bromobenzylglutathione cyclopentyl diester (BrBzGSHCp2). A, BrBzGSHCp2 revealed dose dependent significant inhibition of specific Glo-I activity after 24h treatment of HSC. B, Western blot analysis of α-SMA, TGF-β, NF-κB p65 and Nrf2 in HSC. Quantification (C) showed dose dependent significantly reduced expression of α-SMA, TGF-β and NF-κB after 24h treatment with BrBzGSHCp2 and significant stimulation of Nrf2. Results are expressed as mean ± S.D. of at least three independent experiments. * P<0.05, ** P<0.01, *** P<0.001. (TIFF) [file pone.0171260.s003.tiff]
