# Supplementary material for: Expression of glyoxalase-I is reduced in cirrhotic livers: A possible mechanism in the development of cirrhosis
Source: PLoS One. 2017 Feb 23;12(2):e0171260. doi: 10.1371/journal.pone.0171260 (PMC5322979; doi:10.1371/journal.pone.0171260)
Supplement: S1 File — (DOCX) [file pone.0171260.s004.docx]

**Supplemental material and methods**

Tissue collection

For immunhistochemistry rats were anaesthetized as described above. After perfusion with Krebs-Henseleit solution containing dextrose livers were resected and frozen at -20°C.

Cell lines

Human HSC (HSZ-B-S1) were cultured in DMEM (Sigma-Aldrich, Steinheim, Germany) supplemented with 10% heat-inactivated FCS and 1% PS at 37°C, 10% CO_2_. Mouse TSEC (T-SEC) were cultured in DMEM supplemented with 10% FCS, 0.3% endothelial cell growth support (ECGS, Sigma-Aldrich, St. Louis, USA) and 1% PS at 37°C, 5% CO_2_. Human HepG2 cells (HepG2) were cultured in RPMI supplemented with 10% FCS and 1% PS at 37°C, 5% CO_2_.

Cell lysates and treatment with ethyl pyruvate

For experiments cells (primary cells and cell lines (HSZ-B-S1) were seeded in 100mm dishes (Cellstar Greiner Bio One, Frickenhausen, Germany) at 300.000 cells per dish. At confluence of 70% cells were treated with ethyl pyruvate (EP; 1-20mM) and/or 100ng/ml LPS or BrBzGSHCp_2_ (1-10µM) (all from (Sigma-Aldrich, Steinheim, Germany) in serum-free medium. In case of co-treatment EP was given to the cells prior to LPS-stimulation. After 24h incubation cells were washed twice with PBS (PAA Laboratories GmbH, Pasching, Austria) on ice and lysed with 200µl IPP-lysis-buffer (10mM EDTA (Roth, Karlsruhe, Germany), 1mM EGTA (Roth, Karlsruhe, Germany), 150mM NaCl (AppliChem, Darmstadt, Germany), 1% Triton-X-100 (AlphaAesar, Karlsruhe, Germany), 0.1% PhosStop (Sigma-Aldrich, Germany), 0.1% protease-inhibitor-cocktail (Roche, Mannheim, Germany), pH 7.4). After scraping cells were centrifuged at 13000 x g and 4°C for 15min. Supernatants were collected and stored at -20°C. Protein concentrations were determined using BCA-method following instructions of the manufacturer (Sigma-Aldrich, Steinheim, Germany).

For RNA-Analysis instead of IPP-lysis-buffer 0.5-1ml of trizol (Qiazol, Qiagen, Hilden, Germany) were used. RNA-isolation was adapted according to prior published protocols [29]. RNA-concentrations were determined using photometric measurement.

Immunohistochemistry for Glo-I staining

Rat livers were fixed in 4% formaline (Histofix Roth, Karlsruhe, Germany) for 48h followed by automatic dehydration (microm STP120, Thermo Scientific, Rockford, USA) and embedded in usual histological boxes (Macro Star IV, Engelbrecht, Edermuende, Germany). Sections were made with HM 325 (microm, Walldorf, Germany) and dried over night at room temperature (RT). Descending alcoholic series were followed by demasking and boiled at 250°C. Sections were blocked with 3% H_2_O_2_ for 30min (pharmacy of the University of Halle) and for 1h in 5% BSA (Roth, Karlsruhe, Germany) and 0.5% tween (Roth, Karlsruhe, Germany). Primary antibody (Glo-I (mouse monoclonal IgG, MA1-13029, Pierce, Waltham, Massachusetts, USA)) was incubated over night at 4°C. Sections were washed three times with PBS and incubated with secondary antibody (anti-mouse (IgG-HRP, 715035151, donkey origin, Dianova, Hamburg, Germany)) for 1h at RT. Additional washing was followed by staining with DAB (ImmPACT, Vector, Burlingame, USA) for 2-10min. Staining reaction was stopped in distilled water. For controls, secondary antibodies lacking primary antibody were incubated. Overview pictures and liver sections were analyzed using the Keyence Biozero BZ 8000 microscope with BZ Viewer (Osaka, Japan). At least three livers for each group were analyzed with 10 sections per liver.

RT-PCR

Isolated RNA was performed with DNAse for 30min at 37°C followed by 10min at 65°C in Stop solution (Promega, Madison, USA). Correct DNA-digestion was checked by control-gel. First-strand cDNA was generated from normalized RNA amounts using Oligo-(dT)-primers and the RevertAid Premium First Strand cDNA Synthesis Kit (Fermentas, Rockford, USA) following instructions of the manufacturer. RT-PCR was performed with taq-polymerase (Red PCR master mix, stratec, Berlin, Germany) and specific primer pairs: GAPDH (1177bp fragment; forward: GACCCCTTCATTGACCTC, reverse: GCAATGCCAGCCCCAG; Program: 95°C for 2min, (95°C for 30s, 58°C for 45s, 72°C for 45s) x 32, 72°C for 2min), human Glo-I (921bp fragment; forward: CTTCTGGGGTTTCAATTCCTC, reverse: AATCCATTTCACCCAAAAAGG), mouse Glo-I (940bp fragment; forward: GATTTGGTCACATTGGGATTG, reverse: AGAGAGCATAGGCCAGACTCC), rat Glo-I (914bp fragment; forward: AGCGTCCAGTGGTCTTACTGA, reverse: AAAGCCCAGAAGGTAGCAGAC). For Glo-I primer pairs we used the following PCR program: 95°C for 2min, (95°C for 30s, 56°C for 45s, 72°C for 45s) x 35, 72°C for 2min for human primer pairs and 95°C for 2min, (95°C for 30s, 60°C for 45s, 72°C for 45s) x 35, 72°C for 2min for mouse and rat primer pairs.

ELISA

HSC were seeded in 60mm dishes (Cellstar Greiner Bio One, Frickenhausen, Germany) at 200.000 cells per dish. At 70% confluence HSC were incubated with 1-20mM EP and/or 100ng/ml LPS in serum-free medium for 24h. Supernatants were collected and frozen at -20°C. For TNF-α-ELISA (MyBiosource, San Diego, USA) 200µl supernatant, for collagen-I-ELISA (MyBiosource, San Diego, USA) 40µl supernatant and for α-SMA-ELISA (MyBiosource, San Diego, USA) 40µl supernatant were used following instructions of the manufacturer. For MGO-ELISA 10µl of protein lysates of whole liver tissue or HSC were used per well following instructions of the manufacturer (MBS2605842, MyBiosource, San Diego, USA).
